# Chemotherapeutics-Induced Intestinal Mucositis: Pathophysiology and Potential Treatment Strategies

**DOI:** 10.3389/fphar.2021.681417

**Published:** 2021-05-04

**Authors:** David Dahlgren, Markus Sjöblom, Per M Hellström, Hans Lennernäs

**Affiliations:** ^1^ Department of Pharmaceutical Biosciences, Uppsala University, Uppsala, Sweden; ^2^ Department of Neuroscience, Division of Physiology, Uppsala University, Uppsala, Sweden; ^3^ Department of Medical Sciences, Gastroenterology/Hepatology, Uppsala University, Uppsala, Sweden

**Keywords:** chemotherapeutics-induced mucositis, gastrointestinal physiology, intestinal proliferation, cancer, stem cells, toxicity, mucositis

## Abstract

The gastrointestinal tract is particularly vulnerable to off-target effects of antineoplastic drugs because intestinal epithelial cells proliferate rapidly and have a complex immunological interaction with gut microbiota. As a result, up to 40–100% of all cancer patients dosed with chemotherapeutics experience gut toxicity, called chemotherapeutics-induced intestinal mucositis (CIM). The condition is associated with histological changes and inflammation in the mucosa arising from stem-cell apoptosis and disturbed cellular renewal and maturation processes. In turn, this results in various pathologies, including ulceration, pain, nausea, diarrhea, and bacterial translocation sepsis. In addition to reducing patient quality-of-life, CIM often leads to dose-reduction and subsequent decrease of anticancer effect. Despite decades of experimental and clinical investigations CIM remains an unsolved clinical issue, and there is a strong consensus that effective strategies are needed for preventing and treating CIM. Recent progress in the understanding of the molecular and functional pathology of CIM had provided many new potential targets and opportunities for treatment. This review presents an overview of the functions and physiology of the healthy intestinal barrier followed by a summary of the pathophysiological mechanisms involved in the development of CIM. Finally, we highlight some pharmacological and microbial interventions that have shown potential. Conclusively, one must accept that to date no single treatment has substantially transformed the clinical management of CIM. We therefore believe that the best chance for success is to use combination treatments. An optimal combination treatment will likely include prophylactics (e.g., antibiotics/probiotics) and drugs that impact the acute phase (e.g., anti-oxidants, apoptosis inhibitors, and anti-inflammatory agents) as well as the recovery phase (e.g., stimulation of proliferation and adaptation).

## Introduction

Chemotherapy is in general associated with extensive anti-tumor effects, but also serious adverse effects and long-term safety issues for both cancer patients and healthcare providers (Sougiannis et al., 2021). One of the more common off-target toxicities is chemotherapeutics-induced intestinal mucositis (CIM), which is a complex gastrointestinal (GI) complication. It affects up to 40–100% of all cancer patients dosed with chemotherapeutics, depending drug and dosing regimen (Sonis et al., 2015; Villa and Sonis, 2015). The GI tract is particularly vulnerable to antineoplastic drugs that inhibit cell growth and/or cell division, as the intestinal epithelial cells (IEC) proliferate rapidly and have a complex immunological interaction with the gut microbiota. For instance, antineoplastic drugs such as 5-fluorouracil, methotrexate, irinotecan, and doxorubicin target the vulnerable GI tissue by interrupting DNA synthesis, leading to apoptosis. An inability to resist damage and/or rapidly repair and restore the epithelial barrier function after chemotherapy is detrimental to the cancer patient, as it can result in various pathologies, including inflammation, ulceration, pain, nausea, diarrhea, sepsis, and multiple organ dysfunction and failure (Keefe et al., 2004). In addition to reducing the quality-of-life of these patients, CIM often leads to dose-reduction and subsequent decrease of anticancer effect, sometimes even resulting in death.

Despite substantial improvements in cancer treatments and a continuous decline in its incidence in the population, CIM remains a significant and common clinical challenge in many cancer patients (Henley et al., 2020). Consequently, there is a strong consensus that effective strategies are needed for the prevention and treatment of CIM, including new monotherapies and drug combinations (Scarpignato and Bjarnason, 2019; Dahlgren et al., 2020). Crucial to this endeavor is a better understanding of the pathophysiological factors and adaptive processes involved in the regulation and repair of an injured intestinal epithelium (Odenwald and Turner, 2017). For instance, glucagon-like peptide-1 (GLP-1) and -2 (GLP-2) have a central role in the adaptive recovery response in the small intestine (Hytting-Andreasen et al., 2018; Billeschou et al., 2021). Our contribution to this field is the development of relevant *in vivo* models that provide us with a conceptual and rational approach to treat CIM, coupled with a close and rapid collaboration with clinical partners. This review presents an overview of the functions and physiology of the healthy intestinal barrier followed by a summary of the pathophysiological mechanisms involved in the development of CIM. A literature search was performed using the Pub-Med without any time limit for article inclusion, using the following search words: chemotherapeutics-induced intestinal mucositis, intestinal mucositis, chemotherapeutics gut toxicity, chemotherapeutics gastrointestinal side-effects. Finally, we highlight some of the available pharmacological and microbial interventions (prophylactic, acute, and recovery) that have shown clinical potential, with an emphasis on combination treatments. The main objective of this review was to scrutinize and analyze CIM and to discuss and propose a few novel medical strategies.

## Anatomy and Physiological Functions of the Gastrointestinal Tract

### Anatomy

The morphology of the intestinal barrier varies between regions, but it has a common histology composed of four distinct layers: the mucosa (epithelium, lamina propria, and muscularis mucosae); the submucosa; the muscle layer (circular and longitudinal muscle, and the in-between myenteric nerve plexus); and the serosa. The first barrier between lumen and blood is the mucosal epithelium, which is comprised of columnar IEC covered by a protective mucus layer (Johansson et al., 2013). The IECs are sealed together at the apical surface by tight junction proteins, which form the primary physical barrier to small hydrophilic molecules (approximately less than 250 Da) across the IEC (Fagerholm et al., 1999; Van Itallie and Anderson, 2004). These structurally and biochemically differentiated paracellular regions primarily include tight junctions and anchoring junctions. Tight junctions hold the cells together and form a near leak-proof intercellular seal by fusing adjacent cell membranes, while the anchoring junctions provide essential adhesive and mechanical properties (Andrade et al., 2015). In the small intestine, the mucosa is built up by finger-like villous protrusions that increase the surface area by a factor of about 6 compared to a smooth tube (Helander and Fändriks, 2014). The lamina propria below the IEC layer contains blood vessels, nerve fibers, lymphatic vascular systems, smooth muscle that regulates blood flow and villi movement, and immune cells such as neutrophils, T-regulatory cells, macrophages, and mast cells (about 1 to 10 immune cells per IEC in the epithelium) (Mowat and Agace, 2014). It also contains the most recently identified cells of the innate immune system, the innate lymphoid cells, where they are involved in and coordinate tissue homeostasis during for instance infection, inflammation and cancer by promoting remodeling, healing and repair (Artis and Spits, 2015). The submucosa contains connective tissue with major blood and lymphatic vessels (Bernier-Latmani and Petrova, 2017). The muscle layer contains the submucous plexus, glial cells, cells of Cajal, and circular and longitudinal muscles that control GI movement, while the serosa is mainly composed of connective tissue that supports the GI tract in the abdominal cavity.

The neurons and their nerve fibers in the GI tract are jointly called the enteric nervous system, which is involved in regulation of peristalsis, secretion, digestion and absorption (Furness, 2012). Intestinal microbiota is also sometimes regarded as a part of the GI system, where it is part of a harmonious ecosystem together with the host. It has recently been estimated that the human body hosts up to 10^13^ bacteria, and therefore, about 50% of the cells in our body are non-eukaryotic (Sender et al., 2016). Luminal bacteria and mucosal immune cells show region-related distribution with a higher abundance of bacteria in the distal regions and a more varied immune cell distribution (Mowat and Agace, 2014; Donaldson et al., 2016). Together, they have synergetic roles in maintaining intestinal homeostasis and also the dysregulation associated with intestinal inflammation (Holzapfel et al., 1998).

### Physiological Functions

The primary physiological functions of the GI tract are to digest food and to absorb water and nutrients from the intestines and regulate metabolism. In parallel it acts as a dynamic barrier preventing absorption of peptides/proteins/xenobiotics/toxins and translocation of microbes and viruses into the underlying tissue, organs, and systemic circulation (Marchiando et al., 2010). The intestinal mucosa is thus a selective barrier with the complex task of simultaneously balancing optimal protection against the harsh biochemical and mechanical luminal environment while allowing efficient nutrient absorption (Dahlgren et al., 2014; Ahluwalia et al., 2017). The GI tract is also a highly specialized chemosensory organ, with the capacity to sense nutrients *via* various receptors from the luminal side to optimize and coordinate digestion, metabolism, and absorption of the diet following ingestion of food and fluids, as well as in the defense response to pathogens present in the lumen. The ingestion of a meal starts neural and hormonal signaling from the GI tract in response to gastric distension and the chemical presence of nutrients in the GI lumen (Steinert et al., 2016).

**FIGURE 1:**
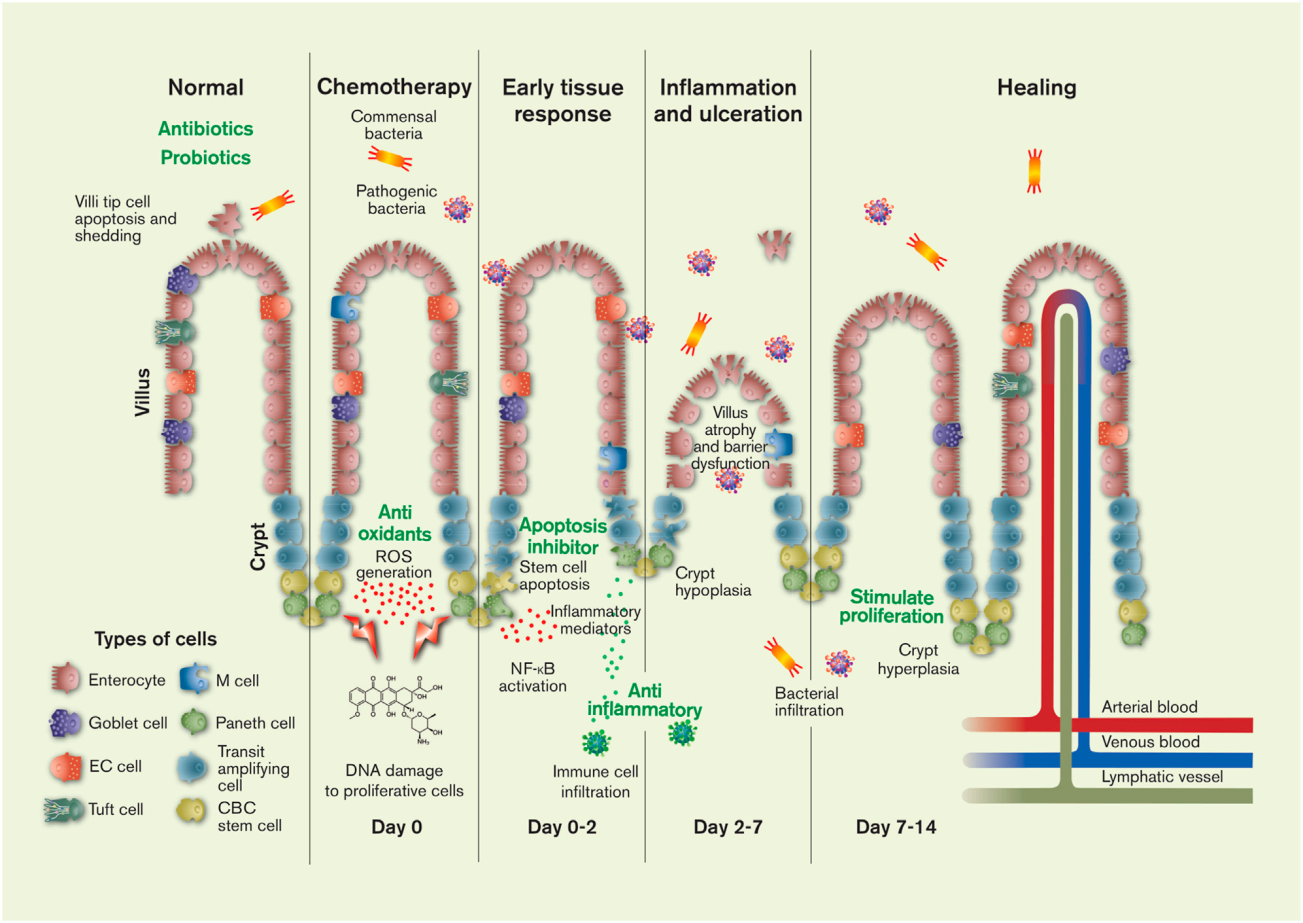
The pathology and timeline of chemotherapeutics-induced intestinal mucositis is primarily related to the effect of cytostatics on stem cells in the proliferation zone of the crypts: crypt base columnar (CBC) stem cells and transit amplifying daughter stem cells. Injury to the DNA of these cells causes apoptosis and initiates of a range of local tissue responses. These include generation of reactive oxygen species (ROS) and inflammation mediators, leading to further injury, inflammation, ulceration, villus and crypt atrophy, and the interstitial infiltration of luminal bacteria (commensal and pathogenic) and immune cells. After about 2 weeks the histology of the intestine is restored in humans (1 week in rodents). The green texts show potential targets for CIM intervention. The figure also shows the six different mature cell types of the intestines, the villi protrusions present in the small intestine, and the lymphatic, venous and arterial vessels. Artwork by Febe Jacobsson. EC = enterochromaffin.

The permeability and health of the intestinal barrier is strictly regulated by a range of neuroendocrine processes, hormones, and luminal stimuli that jointly aim at upholding homeostasis in conjunction with the different IEC (Chelakkot et al., 2018). The intestinal epithelium contains six mature cell types with distinctly different functions: two absorptive IECs (enterocytes and M cells) and four secretory IECs (goblet cells, enteroendocrine cells, Paneth cells, and tuft cells) (Figure 1). The function of the enterocytes is to absorb nutrients, water and ions; they constitute about 80% of the intestinal cells (Gehart and Clevers, 2019). The M-cells are part of the gut-associated lymphoid tissue—the largest immunological tissue in the body—where they allow some uptake of luminal bacteria, thereby triggering or preventing an immunological response depending on the antigen (Ohno, 2016). Thus, the microflora in the intestinal lumen is essential for normal intestinal function and plays an important dynamic role in health and disease progression. Two of the secretory cells primarily secrete into the lumen, where goblet cells secrete protective mucus and the Paneth cells anti-microbial compounds. The other two secretory cells secrete primarily into the interstitium as a response to luminal stimuli. The tuft cells are involved in the defense against parasitic infections. The enteroendocrine cells secrete more than 30 different peptide hormones involved in a range of GI and systemic functions, which makes the gut the largest endocrine system in the body (Gribble and Reimann, 2016).

## Pathophysiology of Chemotherapeutics-Induced Mucositis

### Normal Injury Response and Mucosal Proliferation

**FIGURE 2:**
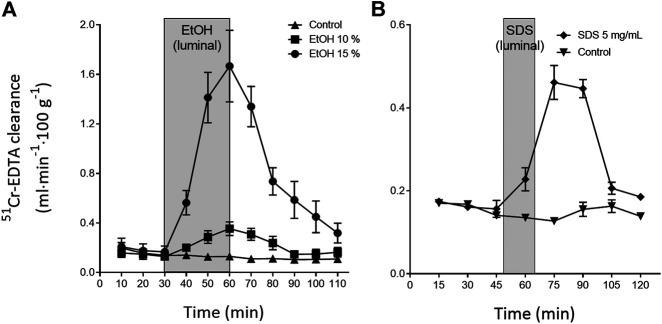
Illustration of the rapid recovery (about 60 min) of the rat small intestinal blood-to-lumen 51Cr-EDTA clearance following local luminal exposure to saline (white area) and two mucosal irritants (grey area): **(A)** ethanol 30 min (Sommansson et al., 2013b) and **(B)** sodium dodecyl sulfate (SDS, anionic surfactant) 15 min (Dahlgren et al., 2018b).

The continuous, everyday mechanical and/or chemical injury to the outer villi sections and epithelium in the lumen is repaired within minutes to hours. This is exemplified in Figure 2, which shows the changes in intestinal permeability of the clinical mucosal integrity marker, 51Cr-EDTA (Dahlgren et al., 2017), following luminal exposure of the rat small intestine to ethanol and sodium dodecyl sulfate. This acute repair process re-establishes the tight junctions thereby restoring the intestinal barrier function and avoiding translocation of harmful luminal bacteria and macromolecules into the underlying mucosa. The repair is also crucial for re-establishing other cellular functions, including water regulation and nutrient absorption. The intestinal integrity and local tissue homeostasis is initially upheld by restitution. This is a process in which IEC at the tip of the villi, and injured IEC, undergo different types of cell death, such as anoikis, apoptosis, necroptosis and pyroptosis (Patankar and Becker, 2020). Dead cells slough off, while neighbouring epithelial cells migrate to close the gap. In healthy intestine, this process occurs without any clinically relevant loss of barrier function (Marchiando et al., 2011; Gehart and Clevers, 2019).

A prerequisite for restitution is a continuous renewal of cells from the lower layer of the epithelium. This renewal takes place in the crypts of Lieberkühn, the proliferative region of the intestinal mucosa. These crypts are positioned at the base of the villus protrusions in the small intestine, and directly on the flat surface of the colon. The crypts are invaginations in the epithelium that are protected from mechanical and chemical injury and pathogens, from the luminal side. Each crypt is thought to contain about 15 crypt base columnar stem cells located at cell positions 1-3 (cp1-cp3) from the bottom, wedged between the Paneth cells that secrete anti-microbial compounds. (Potten et al., 2009) These stem cells divide infinitely once every 24 h to initially form a transit population of rapidly dividing progeny cells. These in turn each divide about six times in total, adding up to about 300 new cells per day per crypt (Gehart and Clevers, 2019). As there are about 4–10 crypts per villus depending on small intestinal region (Keefe, 1998), about 1200–3000 cells are shed every day for each villus.

Generation 1 transit population cells at cell position 4 from the bottom (called +4 cell or cp4) to 3 (cp6) divide rapidly and are uncommitted, whereas the transit population cells are committed from generation 4 (cp7) (Duncan and Grant, 2003; Gehart and Clevers, 2019). These committed cells differentiate into the six distinct intestinal cell types, as discussed previously. With the exception of the Paneth cells that travel to the bottom of the crypts, these post-mitotic cells are pushed outward by the constant renewal in the crypts, and they travel along the villus to finally undergo apoptosis and shedding into the lumen at the tip (Gehart and Clevers, 2019). This way only mature cell types face the harsh environment of the lumen, and only for a relative short time; the epithelial surface of the intestine is renewed about every 3–4 days (Darwich et al., 2014). The sources and essential signalling pathways—and their complex interplay in the determination of cell proliferation and differentiation in the intestinal crypts—have been elegantly illustrated by Gehart and Clevers (Gehart and Clevers, 2019). In principal these processes are balanced by two opposing top-to-bottom crypt gradients. In the one gradient, WNT secreted by the Paneth cells and mesenchymal cells in the crypt bottom maintain stem cell function. In the other gradient, bone morphogenetic proteins—secreted by mesenchymal cells higher up in the crypts—counteract the effect of WNT to induce cell maturation. Wnt signaling is a highly conserved pathway that plays principal regulatory roles in many developmental and biological processes. Besides its crucial role in tissue homeostasis, Wnt signaling is also found to be activated aberrantly in many human diseases, including cancers and metabolic disorders (Novellasdemunt et al., 2015).

### Mechanisms for Chemotherapeutics-Induced Mucositis

The DNA of crypt stem cells is well protected from the luminal environment. Fluid flows steadily outwards and interspaced Paneth cells secrete antimicrobial products, making crypts essentially a sterile environment (Nylander and Sjöblom, 2007; Wehkamp and Stange, 2020). However, injury to the DNA in stem cells may arise from events like radiation and cytostatics, causing the cells to go into apoptosis as well as other types of cell death. Still, even when the stem cell pool is completely wiped out it is replenished within a few days. This is possible primarily because initial generations of progeny cells may revert back to the parent stem cell type in the crypt when these are lost. However, others claim that also more committed cells may de-differentiate and repopulate the crypt stem cells upon injury (Buczacki et al., 2013; Yan et al., 2017).

One key issue with chemotherapy is what happens when the cell mitosis and amplification processes in the cryptal stem cells and progenitor cells are compromised by apoptosis. The degree of apoptosis and the local cryptal variations differ depending what cytostatic drug that is used (Ijiri and Potten, 1983). Regardless, normal cell maturation and regeneration of the epithelium is impaired, which means that the continuous (normal) shedding of apoptotic IECs at the tip of the villi is unaccompanied by adequate cellular renewal. In addition, antineoplastic drugs may also be harmful to non-dividing cell populations in the intestine, potentiating any negative effects of an altered cryptal cell renewal. For example, the cytostatic doxorubicin (DOX) is associated with both production of reactive oxygen species and mitochondrial dysfunction (van der Zanden et al., 2020).

Sonis et al. have proposed a general five-stage model for the development of CIM over time (Figure 1): 1) initiation, 2) signalling activation and primary damage response, 3) amplification of biological pathways, 4) tissue inflammation and ulceration, and 5) healing (Sonis, 2009; Al-Dasooqi et al., 2013). The *initiation phase* is characterized both by direct DNA injury and the generation of reactive oxygen species. The *primary damage response* starts within seconds of DNA strand breaks and the reactive oxygen species *activate signalling* factors such as Wnt/β-catenin, p53, caspase-1/3, Bcl-2 and NF-κB, and their associated pathways (Bowen et al., 2006; Sukhotnik et al., 2014; Bowen et al., 2019). These effects jointly cause death to the intestinal stem cell population and subsequent breakdown of the intestinal barrier. NF-κB is especially well studied in CIM, because it plays a fundamental role in pathogenesis by regulating a range of cytokines (e.g., TNF-α, IL-6, IL-1, IL-18, and IL-33), stress responders, cell adhesion molecules, as well as apoptosis in normal cell populations (Ribeiro et al., 2016). Many of these effects leads to *signalling amplification*, whereby the positive and negative feedback responses of the initial factors affect the local tissue in a complicated biochemical interplay. For instance, NF-κB activates TNF-α release, which in turn activates more NF-κB. The overall effect of the overwhelming biochemical response is mucosal *inflammation and ulceration*, characterized by an ablation of the epithelial villi, a disruption of IEC adhesion, and an increased translocation of luminal components and immune cells into the lamina propria. This cascade of events leads to even more inflammation. The final stage is the spontaneous *healing* phase in which normal epithelial proliferation, migration, differentiation and maturation are restored.

The whole alimentary tract is formed from the same structure in the embryo (Stringer et al., 2009), and any effects of chemotherapy should be similar in all regions (oral cavity, stomach, small and large intestine) as the same genes are activated (Yeoh et al., 2007). Nonetheless, there are important physiological and anatomical differences. The mouth and small intestine seem to be most affected by mucositis, and have therefore been the regions most studied (Keefe et al., 2004). The dissimilarity in injury has been attributed to the different regional expression of pro- and anti-apoptotic factors, such as Blc-2, which amplifies apoptosis in the small intestinal crypts (Bowen et al., 2005). Spontaneous apoptosis is 10 times more common in the small intestine than the large intestine, and the small intestine is therefore, not surprisingly, more vulnerable to mucositis induced by chemotherapeutics and radiotherapy (Bowen et al., 2006). The lower apoptosis frequency in the large intestine also contributes to the higher incidence of cancers in the lower compared to the upper intestinal tract.

**FIGURE 3:**
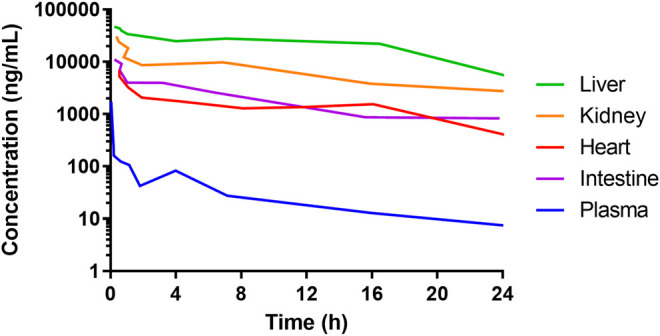
Concentrations of doxorubicin in plasma and liver, heart, kidney, and intestines of mice following 5 mg/mL intravenous administration of a solution. Data from Luo et al. (2017). The high concentration of doxorubicin in all the organs shows that the side-effects of many anti-cancer drugs are not ubiquitously dose-dependent. Rather, they are associated with the tissue-specific cell proliferation rate. This is why cancer tissue and healthy intestinal tissue are typically heavily affected.

The time from drug exposure to the epithelial effects varies for different species, doses, administration routes and type of chemotherapeutics, and partly follows species-specific differences in crypt turnover. For instance, after an intravenous dose of DOX, the concentration in the intestine is about 100 times higher than in plasma in animals and humans (Luo et al., 2017; Lee et al., 2020). Although the DOX concentrations in the intestines might be similar as in the liver, kidney, and heart, they cause greater damage to the IECs because these cells have a rapid and extensive proliferation (Figure 3) (Luo et al., 2017). In mouse and rat, the cellular apoptosis in the crypts peaks at about 6–24 h after DOX administration (Thakkar and Potten, 1992), whereas the maximum effects of the villi height and crypt depth peaks at about 72–96 h (Dekaney et al., 2009). This is also the same time interval after DOX treatment at which the cellular renewal process is peaking in the crypts (Dekaney et al., 2009). A complete recovery of the mucosa and its function are restored after about one week in mouse and rat. In humans, these processes are similar to the rodent models, but the peak times are different and the overall time to recovery is about twice as long (Keefe et al., 2004).

CIM not only affects the stem cell population. It also has a complex interplay between the many mucosal cell types (e.g., IEC, immune cells, mesenchymal cells) in the different intestinal compartments (e.g., villus, crypts, intra and extracellular, mucus). These cell types and compartments are important in the injury and healing following cytostatics treatment. For instance, germ-free mice experience the same amount of DOX-induced increase in cryptal apoptosis as normal mice, but the overall intestinal mucosal injury is greater in the normal mice (Rigby et al., 2016). Single intraperitoneal injection of methotrexate (20 mg/kg) to Sprague–Dawley rats (200–250 g) causes severe enterocolitis and death (Mao et al., 1996). However, oral administration of lactobacilli to the treated rats significantly improves their intestinal nutritional status and dynamic barrier function, reduces the number of enteric pathogenic bacteria, and most likely explains the reduction of the bacterial translocation and endotoxemia.

This illustrates the symbiotic interplay between microbiota and the activation of the immune system in maintaining intestinal homeostasis. This is further exemplified by the role of the TLR receptors 2 and 9 that are expressed on a range of intestinal cell types. These receptors recognize bacterial epitopes and determine different responses to commensal and other intestinal bacteria. Mice lacking these receptors display less CIM, most likely as a result of a downregulation of intestinal apoptosis.(Kaczmarek et al., 2012) The extracellular matrix is also important for maintaining tissue morphology and healing. The cancer drug irinotecan is known to affect extracellular matrix protein expression, which contributes to cell cytostasis and apoptosis followed by an increase in collagen deposits partly attributed to changes in the expression of metalloproteinases (Al-Dasooqi et al., 2010; Al-Dasooqi et al., 2011). Furthermore, after cytostatics treatment, it is fundamental for mucosal health that the protective epithelial mucus layer is rebuilt by the mucins. These mucins are involved in cell proliferation, the inhibition of apoptosis, and the overall severity of CIM (Thorpe, 2019).

The multitude of parameters involved in CIM, and our improved understanding of its pathophysiology, give rise to many possible targets for various treatment strategies. Below and in Table 1 follows a summary of some interesting past and recent studies and potential targets.

**TABLE 1:** Potential future treatment options and some examples of specific interventions for CIM. Please see the text for a more detailed description of the proposed treatment strategies.

Treatment options	Examples	Mechanisms
Microbiota	Antibiotics	Reduces pathogenic intestinal bacteria and mucosal infections
	Dihydrotanshinoneon	Restores normal gut microbiota
	Probiotics and fecal microbiota transplantations	Reduces diarrhea, reduce pathogenic bacteria, modulating inflammatory response
Anti-oxidants	Amifostine, melatonin	Detoxifies reactive metabolites of chemotherapeutic agents and scavenges free radicals
Mucosal barrier regulators	Melatonin	Reduces basal and GI injury increases in intestinal permeability
Anti-inflammatory agents	Misoprostol, COX-2 inhibitors	Reduces inflammatory response and propagation
Anti-apoptotic agents	IL-1 receptor antagonist, β-arrestins	Suppression of crypt cell death
Incretins	GLP1 and GLP2	Stimulate growth, promote healing and inhibits epithelial apoptosis
Growth hormones	Keratinocyte growth factor	Stimulates proliferation

## Possible Treatment Options for CIM

There is an unmet need to identify and develop efficient drug treatments for GI toxicities caused by chemotherapeutics (Stringer et al., 2009; Sougiannis et al., 2021). The overall aims of any intervention are to reduce the GI related symptoms experienced by cancer patients—this would relieve suffering, enable dose escalation, or avoid dose de-escalation. Interventions can include prophylactic treatments such as probiotics and antibiotics to prepare the GI tract. They may also include anti-oxidants, anti-inflammatory drugs, and apoptosis inhibitors during cytostatics treatment to alleviate some of the immediate toxicities and associated effects. Lastly, treatments such as incretins and growth hormones can be used after cytostatic dosing to benefit the mucosal adaptation and proliferation processes after injury. This section discusses some promising interventions that can be deployed in each of the three stages. Finally, we highlight the usefulness of combining treatment options to tackle CIM from multiple angles.

### Microbial and Anti-microbial Treatments

The microbiota can have both detrimental and supportive effects on GI homeostasis and health (Benno et al., 2019). This also holds true for CIM, where luminal bacteria are involved in the regulation of intestinal barrier functions, maintenance of selective intestinal permeability, inflammation and innate immune response, repair mechanisms, cell apoptosis, and oxidative stress (Prisciandaro et al., 2011). The direct or indirect effects of cytostatics on gut microflora dysbiosis also impact the clinical manifestations of CIM, where they contribute to the development of bacteremia and diarrhea. Accordingly, there is an abundance of preclinical CIM rodent models that have reported positive effects of antibiotics (Hamouda et al., 2017), fecal transplantations (Chang et al., 2020), and probiotics (Yeung et al., 2015; Quaresma et al., 2020). In a mouse model, CIM toxicity has been reduced with dihydrotanshinoneon (a liposoluble plant extract) that restores normal gut microbiota (Wang et al., 2020). In patients undergoing GI surgery, a changed microbial intestinal flora in combination with an altered barrier function may progress to an enhanced inflammatory response. Here probiotics may reduce pathogenic bacteria (Jeppsson et al., 2011). Still, in spite of the vast literature supporting the use of treatments directly or indirectly targeting the luminal microbiota, treatments for CIM that alter the GI microbiome have largely failed in the clinic (Touchefeu et al., 2014), Consequently, there is a need to improve and establish the most suitable composition of type of probiotic bacteria, and its dose and length of treatment. Of special interest is the possibility of combining interventions, such as pre-treatment with antibiotics that target bacterial populations with noxious membrane effects, and beneficial probiotic/fecal microbiota transplantations.

### Anti-Oxidants and Mucosal Barrier Regulators

Amifostine is a phosphorylated aminothiol prodrug, which is rapidly hydrolysed *in vivo* by alkaline phosphatase to the active cytoprotective thiol metabolite, WR-1065. This metabolite has a terminal half-life of 90 min (Ranganathan et al., 2018). Intracellularly WR-1065 detoxifies reactive metabolites of chemotherapeutic agents and scavenges free radicals (Bensadoun et al., 2006); it may also accelerate DNA repair and inhibit apoptosis. As such, intravenous administration of WR-1065 may protect intestinal epithelium and connective tissue from various anti-tumor treatments (Grdina et al., 2000). It reduces DOX-induced CIM in rats (Jaćević et al., 2018) and methotrexate-induced CIM in mice, an effect that is potentiated by co-administration of calcium folinate (Chen et al., 2013) The FDA indication for amifostine refers to xerostomia prophylaxis in post-operative head-neck-cancer patients treated with radiotherapy (Antonadou et al., 2002); however, the data are conflicting about its value in oral CIM prevention (Nicolatou-Galitis et al., 2013). Unfortunately, significant side-effects (mainly nausea and hypotension) limit its clinical use.

Melatonin, a serotonin derivative, is a hormone that controls the sleep–wake cycle and is primarily released by the pineal gland at night (Auld et al., 2017). Melatonin is also synthesized and released by the enterochromaffin cells in the intestine, where it binds to the melatonin membrane receptors MT1 and MT2, and to the cytosolic MT3 receptor (Soták et al., 2006; Söderquist et al., 2015). It also scavenges free radicals (Hardeland and Pandi-Perumal, 2005). In rats and mice, melatonin reduces basal intestinal permeability through an inhibitory nicotinic receptor-mediated neural pathway (Sommansson et al., 2013a). This mitigates ethanol-, chemical-, and radiation-induced intestinal damage (Monobe et al., 2005; Sommansson et al., 2013a; Chamanara et al., 2019), as well as methotrexate-induced oxidative stress and injury (Kolli et al., 2013). Clinical trials with melatonin also report positive effects in irritable bowel syndrome and inflammatory bowel disease (Rakhimova, 2010; Siah et al., 2014). In summary, melatonin has a potent effect on mitigating mucosal injury. It should therefore be investigated for limiting CIM, in particular in synergism with other treatments. For example, melatonin dosed with misoprostol abolishes unselective surfactant-induced intestinal injury in rat (Dahlgren et al., 2020).

### Anti-Inflammatory and Anti-apoptotic Agents

Pro-inflammatory cytokines, such as IL-1, are involved in the progression of CIM (Kanarek et al., 2014), and their natural antagonists are released upon intestinal injury (Daig et al., 2000). As such, the IL-1 receptor antagonist is repeatedly shown to reduce 5-fluorouracil-induced CIM in mice (Wu et al., 2010; Wu et al., 2011). These effects are attributed to reduced crypt cell death by suppression of p53-dependent apoptosis caused by cytotoxic treatments (Wang et al., 2015). Other mediators in cell apoptosis are β-arrestins that suppress p53 levels (Hara et al., 2011). For example, mice deficient in β-arrestin1 experience increased cell death and injury following cytostatics (Zhan et al., 2016). Other ways to reduce caspase-3 activated cells and apoptosis in mice after 5-fluorouracil-induced mucositis include: a serotonin-receptor antagonist (Yasuda et al., 2013); andrographolide (an herbal extract) (Xiang et al., 2020); and armillariella oral solution (a fungus extract) (Wenqin et al., 2019). These preclinical studies show the potential in targeting cell apoptosis pathways to limit mucosal manifestations and complications in CIM.

The prostaglandin E_1_ analogue, misoprostol, is an agonist of G protein-coupled prostaglandin E receptors 1-4 that are involved in epithelial homeostasis and protect against intestinal mucosal damage (Abramovitz et al., 2000). Misoprostol protects by regulating gastric acid and mucus secretion, pro-inflammatory cytokine production, and by activating adaptive cell survival pathways through selective gene repression and splicing (Davies et al., 2001; Field et al., 2018). It is therefore used for the prevention of nonsteroidal anti-inflammatory drug-induced mucosal erosions and ulcers (Graham et al., 1988; Sugimoto and Narumiya, 2007). It is effective at reducing radiation induced injury in animal models (Hanson et al., 1988; Delaney et al., 1994), but its clinical use for treating radiotherapy-induced intestinal and oral mucositis have generated conflicting results, both positive (Hanson et al., 1995) and negative (Duenas-Gonzalez et al., 1996) outcomes. Still, the abundant clinical and preclinical data supporting its cytoprotective effects for a range of GI inflammation and injury models make it a promising drug for further investigations with CIM.

Cyclooxygenase (COX) 1 and 2 are enzymes involved in the formation of prostanoids, which are involved in numerous physiological processes including inflammation (Dahlgren et al., 2018a). COX-1 is expressed and produced constitutively whereas COX-2 production (prostaglandin E2) is induced at sites of inflammation by pro-inflammatory agents (e.g., IL-1, TNF-α) and transcription factors (e.g., NF-κB) (Turini and Dubois, 2002). The involvement of these mediators in the progression of CIM has led several studies to explore the possible contribution of COX-2 to the amplification phase rather than the acute phase of CIM (Sonis et al., 2004). Accordingly, selective COX-2 inhibition is reported to reduce the overall histopathological changes and/or diarrhoea induced by various cytostatics in rodent models [e.g., 5-fluorouracil (De Miranda et al., 2020) and irinotecan (Javle et al., 2007)]. The same treatments have also been used in the clinics for radiation and chemotherapeutics-induced oral and intestinal mucositis, but with mixed results (Javle et al., 2007; Lalla et al., 2014). Overall, the overlap between effectors in CIM and in inflammatory induction of COX-2 make them a possible adjuvant treatment target.

### Incretins and Growth Hormones

Endogenous glucagon-like peptides GLP-1 and GLP-2 are released by the enteroendocrine L-cells into the lamina propria and circulation following oral nutrient ingestion. They stimulate growth, increase absorption, promote healing, maintain intestinal epithelial integrity, and potentially have anti-inflammatory activity (Drucker et al., 1996; Drucker and Yusta, 2014; Ebbesen et al., 2019; Billeschou et al., 2021). The positive effect of luminal food on epithelial growth is also why enteral feeding should be maintained during chemotherapy (Bengmark and Jeppsson, 1995). Plasma levels of GLP-1 correlate with the systemic inflammation in cancer patients receiving chemotherapy; plasma GLP-2 concentrations are significantly elevated 2–5 days following induction of CIM in rats and mice (Kissow et al., 2012; Hytting-Andreasen et al., 2018; Ebbesen et al., 2019). Besides the enhancement of proliferation, exogenous GLP-2 inhibits epithelial apoptosis (Tsai et al., 1997; Boushey et al., 2001). Other studies show GLP-1 and 2 to be central in the adaptive recovery response in the small intestine following CIM (Kissow et al., 2013; Hytting-Andreasen et al., 2018; Billeschou et al., 2021). Thus, GLP-1 and 2, their analogues (semaglutide/exenatide and teduglutide/glepaglutide), or inhibition of their enzymatic-mediated degradation (DPP-IV inhibitors) have great promise for improving mucosal regeneration after CIM, in part by reducing chemotherapy-induced apoptosis (Boushey et al., 2001). GLP-2 analogues also have clinical potential when the integrity or absorptive function of the intestinal mucosa is affected (Salaga et al., 2018).

Another interesting growth factor is the keratinocyte growth factor (KGF), a protein in the fibroblast growth factor family. KGF is a small signalling molecule that binds to fibroblast growth factor receptor 2b which is expressed in the intestine (Song et al., 2020). KGF stimulates proliferation and increases the overall weight of the intestine (Housley et al., 1994). It has been evaluated in rodent models of CIM, but effects have been both positive (Farrell et al., 1998) or absent (Gibson et al., 2002). A human recombinant version of KGF, palifermin, is the only approved (oral) drug treatment for CIM today. As an injection drug, it is used for treating severe oral mucositis in patients receiving myeloablative radiochemotherapy (Nasilowska-Adamska et al., 2007). The cytoprotective effects of palifermin could be expanded to include other indications (Vadhan-Raj et al., 2013).

### Combination Treatments

Despite decades of experimental and clinical investigations of CIM, no effective therapeutic interventions are available today for treating it (Ribeiro et al., 2016; Wardill et al., 2019). What treatments that do exist aim at reducing secondary complications to treatment, such as pain and diarrhoea. Consequently, no single treatment to date substantially transforms the clinical management of CIM, despite numerous promising preclinical investigations. This cements the fundamental role of stem cell proliferation in mucosal health and homeostasis, and suggests that its disturbance by chemical agents is so fundamental that no single intervention can readily compensate. Unless any novel breakthrough occurs in this regard, it is our belief that combinations of treatments are necessary to generate any substantial clinical breakthrough in CIM management. A few example of successful additive combinations treatments for alleviating CIM in preclinical models include GLP 1 and 2 (Hytting-Andreasen et al., 2018), troxerutin and celecoxib (De Miranda et al., 2020), amifostine and calcium folinate (Chen et al., 2013). The combinations with the most potential to be successful (high positive ratio of effect/safety) remain to be investigated, validated, and established. An optimal intervention would likely target the pre-treatment phase of CIM with prophylactics (e.g., antibiotics/probiotics), the acute phase with anti-oxidants and anti-inflammatory agents, and the recovery phase, by stimulation of cell proliferation.

## Conclusions

Gastrointestinal injury and symptoms following chemotherapy in cancer patients remains an unsolved clinical issue. As there are currently no effective treatment options for chemotherapeutics-induced intestinal mucositis, there is no way to help these patients other than by lowering the dose of the cytotoxic drug. However, recent progress in the understanding of the molecular and functional pathology of CIM provides many new potential targets and treatment opportunities. We believe that the best possibility for success is to pursue combination treatments that target different aspects of the complex pathological mechanisms involved in intestinal mucositis.
